# Supplementary material for: Controlling for Structural Changes in the Workforce Influenced Occupational Class Differences in Disability Retirement Trends
Source: Int J Environ Res Public Health. 2019 Apr 30;16(9):1523. doi: 10.3390/ijerph16091523 (PMC6539389; doi:10.3390/ijerph16091523)
Supplement: Supplementary file 1 [file ijerph-16-01523-s001.pdf]

SUPPLEMENTARY MATERIAL CAPTIONS

Table S1. Distribution of education (%) in the original and matched sample by gender, occupational class and calendar year.

	Original sample			Matched sample		
	Primary	Secondary	Tertiary	Primary	Secondary	Tertiary
Men						
Upper-level non-manual employees						
2007	4.4	14.3	81.3	4.4	15.5	80.1
2010	4.1	14.9	81.0	4.2	14.8	81.0
2013	3.5	13.3	83.2	3.8	13.8	82.4
Lower-level non-manual employees						
2007	13.8	39.2	47.0	14.9	47.2	37.9
2010	12.1	41.6	46.3	12.5	42.8	44.7
2013	10.1	41.2	48.7	11.9	42.2	45.9
Skilled manual workers						
2007	26.0	68.9	5.1	22.9	72.5	4.6
2010	22.7	71.6	5.7	22.8	71.8	5.4
2013	20.6	73.7	5.7	23.1	71.6	5.3
Unskilled manual workers						
2007	35.7	56.5	7.8	30.9	60.8	8.3
2010	31.6	59.6	8.8	32.1	59.4	8.5
2013	29.2	61.9	8.9	32.2	59.7	8.1
Self-employed farmers and agricultural workers						
2007	30.1	58.1	11.8	28.9	62.3	8.8
2010	25.3	61.3	13.4	25.9	61.1	13.0
2013	23.0	61.5	15.5	25.2	61.1	13.7
Other self-employed						
2007	25.6	46.8	27.6	23.6	50.3	26.1
2010	22.8	48.8	28.4	23.4	49.3	27.3
2013	20.3	50.4	29.3	22.9	49.6	27.5
B) Women						
Upper-level non-manual employees						
2007	2.3	9.7	88.0	2.0	10.3	87.7
2010	1.9	8.8	89.3	2.0	9.2	88.8
2013	1.4	7.4	91.2	1.9	8.6	89.5
Lower-level non-manual employees						
2007	12.2	44.1	43.7	10.4	48.2	41.4
2010	9.5	44.6	45.9	9.2	46.0	44.8
2013	7.2	44.4	48.4	9.0	46.2	44.8
Skilled manual workers						
2007	32.3	56.6	11.1	25.2	62.6	12.2
2010	26.8	60.1	13.1	25.3	62.4	12.3
2013	23.2	62.3	14.5	26.8	60.4	12.8
Unskilled manual workers						

2007	33.3	60.7	6.0	25.4	66.2	8.4
2010	28.4	63.4	8.2	26.5	64.7	8.8
2013	24.6	66.3	9.1	28.1	63.7	8.2
Self-employed farmers and agricultural workers						
2007	20.2	59.0	20.8	17.5	63.3	19.4
2010	16.2	59.7	24.1	18.0	61.6	20.4
2013	12.7	60.2	27.1	16.0	61.9	22.1
Other self-employed						
2007	16.7	47.4	35.9	12.5	49.8	37.8
2010	13.3	47.7	39.0	13.3	47.7	39.0
2013	11.3	46.3	42.4	13.6	47.7	38.7

Table S2. Year-specific distribution (%) of the matched study population over the study period by background characteristics among (A) men and (B) women

				Change (percentage points)		
	2007	2010	2013	2007-2010	2010-2013	2007-2013
	N	N	N			
A) Men	456365	456365	456365			
Age						
30-34	15.6	16.4	16.7	0.8	0.3	1.1
35-39	16.5	15.1	16.5	-1.4	1.4	0.0
40-44	18.3	17.9	16.3	-0.4	-1.6	-2.0
45-49	17.7	18.4	18.0	0.7	-0.4	0.3
50-54	17.1	17.1	17.2	0.0	0.1	0.1
55-59	14.8	15.2	15.3	0.4	0.1	0.5
Occupational class						
Upper-level non-manual employees	27.4	23.9	22.2	-3.5	-1.7	-5.2
Lower-level non-manual employees	24.3	23.9	25.3	-0.4	1.4	1.0
Skilled manual workers	27.9	29.5	29.2	1.6	-0.3	1.3
Unskilled manual workers	4.7	6.2	6.6	1.5	0.4	1.9
Self-employed farmers and agricultural workers	5.9	5.2	4.6	-0.7	-0.6	-1.3
Other self-employed	9.7	11.3	12.2	1.6	0.9	2.5
Major region						
Southern Finland	30.6	30.1	31.4	-0.5	1.3	0.8
Western Finland & Åland	21.7	21.6	21.3	-0.1	-0.3	-0.4
Eastern Finland	25.0	25.4	25.0	0.4	-0.4	0.0
Northern Finland	22.7	22.9	22.3	0.2	-0.6	-0.4
Employment sector						
Private, domestic	67.1	66.9	67.3	-0.2	0.4	0.2
Private, foreign	10.2	9.7	9.2	-0.5	-0.5	-1.0
Public	22.7	23.4	23.5	0.7	0.1	0.8
Physically heavy work	30.7	31.7	30.1	1.0	-1.6	-0.6
Mean days in full work duties						
-2 year	292		286		281	
-1 year	288		288		279	
Mean days on full sickness absence						
-2 year	4.0		3.3		3.1	
-1 year	2.9		2.6		2.6	
Mean days in unemployment						
-2 year	6.2		8.7		6.5	
-1 year	9.7		8.1		10.3	
B) Women	429442	429442	429442			

Age						
30-34	13.7	14.7	15.1	1.0	0.4	1.4
35-39	15.6	14.4	15.2	-1.2	0.8	-0.4
40-44	18.3	17.3	15.8	-1.0	-1.5	-2.5
45-49	18.5	19.1	18.5	0.6	-0.6	0.0
50-54	18.3	18.6	18.2	0.3	-0.4	-0.1
55-59	15.5	15.9	17.1	0.4	1.2	1.6
Occupational class						
Upper-level non-manual employees	26.5	22.4	21.8	-4.1	-0.6	-4.7
Lower-level non-manual employees	54.6	56.8	57.3	2.2	0.5	2.7
Skilled manual workers	4.7	4.9	5.0	0.2	0.1	0.3
Unskilled manual workers	7.4	8.4	7.8	1.0	-0.6	0.4
Self-employed farmers and agricultural workers	1.5	1.5	2.0	0.0	0.5	0.5
Other self-employed	5.4	5.9	6.1	0.5	0.2	0.7
Major region						
Southern Finland	32.9	32.2	32.2	-0.7	0.0	-0.7
Western Finland & Åland	21.6	22.3	21.9	0.7	-0.4	0.3
Eastern Finland	23.6	23.4	24.1	-0.2	0.7	0.5
Northern Finland	21.8	22.0	21.9	0.2	-0.1	0.1
Employment sector						
Private, domestic	44.6	45.0	45.2	0.4	0.2	0.6
Private, foreign	10.0	9.2	8.6	-0.8	-0.6	-1.4
Public	45.4	45.8	46.2	0.4	0.4	0.8
Physically heavy work	21.5	23.7	22.5	2.2	-1.2	1.0
Mean days in full work duties						
-2 year	296	291	284			
-1 year	288	288	279			
Mean days on full sickness absence						
-2 year	5.5	4.2	3.8			
-1 year	4.3	3.6	3.3			
Mean days in unemployment						
-2 year	10.9	10.3	9.0			
-1 year	16.7	13.5	13.4			

A) All-cause disability retirement. Men.

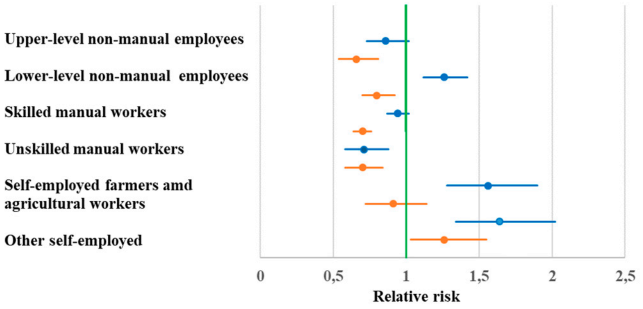

B) All-cause disability retirement. Women.

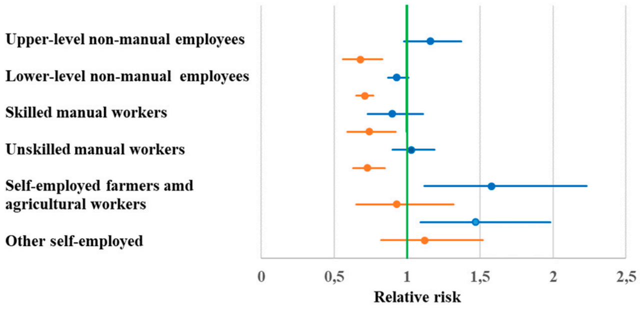

C) Disability retirement due to MSD. Men.
Women.

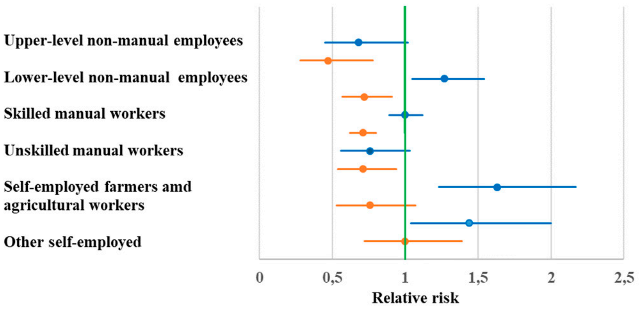

D) Disability retirement due to MSD.

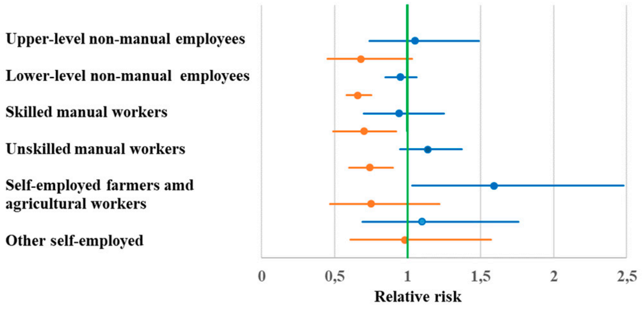

E) Disability retirement due to mental disorders. Men.
disorders. Women.

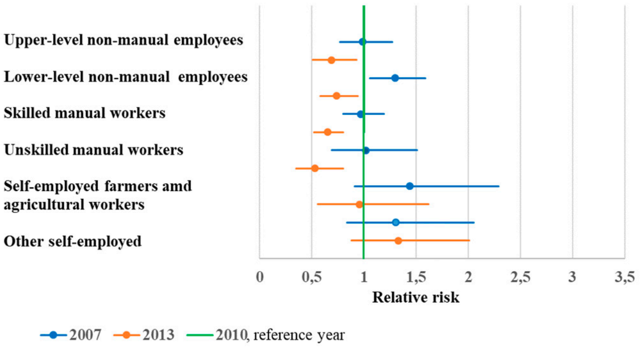

F) Disability retirement due to mental

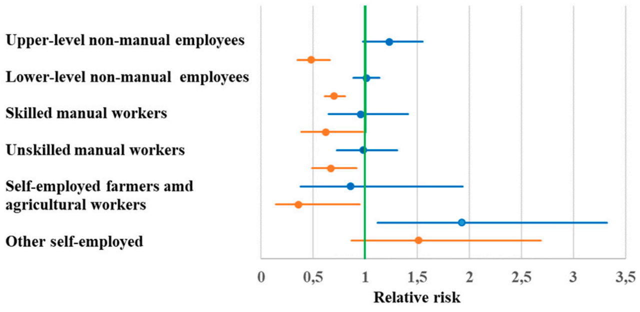

Figure S1. Age adjusted hazard ratios (HR) and their 95% confidence intervals (95% CI) for all-cause and cause-specific disability retirement by observational year and occupational class among 30–59 years old men and women. Reference group: year 2010 (HR=1.00). (A–B) all-cause disability retirement, (C–D) disability retirement due to musculoskeletal diseases (MSD), and (E–F) disability retirement due to mental disorders. Location of the blue line on the right side from the reference line (green line) indicates the risk of disability retirement in year 2007 was lower than in 2010. Location of the orange line on the left side from the reference line (green line) indicates the risk of disability retirement in year 2013 was lower than in 2010.
